# Supragingival Plaque Microbiomes in a Diverse South Florida Population

**DOI:** 10.3390/microorganisms12091921

**Published:** 2024-09-21

**Authors:** Sharlene Demehri, Saynur Vardar, Cristina Godoy, Jose V. Lopez, Paisley Samuel, Toshihisa Kawai, Andrew T. Ozga

**Affiliations:** 1 Department of Periodontology, College of Dental Medicine, Nova Southeastern University, Fort Lauderdale, FL 33328, USA; sd1977@mynsu.nova.edu (S.D.); svardarsengul@nova.edu (S.V.); 2 Department of Oral Science and Translational Research, College of Dental Medicine, Nova Southeastern University, Fort Lauderdale, FL 33328, USA; 3 Department of Public Health, Dr. Kiran C. Patel College of Osteopathic Medicine, Nova Southeastern University, Fort Lauderdale, FL 33328, USA; 4 Department of Biological Sciences, Halmos College of Arts and Sciences, Guy Harvey Oceanographic Center, Nova Southeastern University, Fort Lauderdale, FL 33328, USAps1117@nova.edu (P.S.)

**Keywords:** oral microbiomes, human microbiota, dental bacteria, supragingival plaque, periodontal disease, oral health, 16S rRNA gene sequencing, next generation sequencing

## Abstract

Trillions of microbes comprise the human oral cavity, collectively acting as another bodily organ. Although research is several decades into the field, there is no consensus on how oral microbiomes differ in underrepresented groups such as Hispanic, Black, and Asian populations living in the United States. Here, using 16S ribosomal RNA sequencing, we examine the bacterial ecology of supragingival plaque from four quadrants of the mouth along with a tongue swab from 26 healthy volunteers from South Florida (131 total sequences after filtering). As an area known to be a unique amalgamation of diverse cultures from across the globe, South Florida allows us to address the question of how supragingival plaque microbes differ across ethnic groups, thus potentially impacting treatment regiments related to oral issues. We assess overall phylogenetic abundance, alpha and beta diversity, and linear discriminate analysis of participants based on sex, ethnicity, sampling location in the mouth, and gingival health. Within this cohort, we find the presence of common phyla such as Firmicutes and common genera such as Streptococcus. Additionally, we find significant differences across sampling locations, sex, and gingival health. This research stresses the need for the continued incorporation of diverse populations within human oral microbiome studies.

## 1. Introduction

The oral microbiome has the second highest concentration of prokaryotes within the body (after the gastrointestinal tract) and contains upwards of 700 unique species [1]. The presence of these microbes that colonize both hard and soft tissues within the mouth have been linked to a multitude of host variations [2]. These microbes are also implicated as risk factors for systemic diseases throughout the body, including inflammatory bowel disease, cardiovascular disease, Alzheimer’s disease, rheumatoid arthritis, and various cancers [3]. There are a number of factors that are thought to impact the diversity and abundance of microbiota present within this ecosystem, including demographic information, diet, environmental stressors, and host genetics [4,5,6,7]. We are still learning more about the function of the genes of these microbes as a result of these variables across individuals and how they contribute to an overall ‘core’ human microbiome [8].

Two strong drivers of microbial diversity, in addition to health status, are thought to be the ethnicity of an individual and, a more methodological aspect, the precise location of sampling within the oral cavity. Studies have identified specific taxa that are more abundant within people of certain ethnic groups, which could be caused by numerous lifestyle factors [9,10,11]. These studies are incredibly beneficial as there has been a push in the last decade to include individuals from a wide range of ethnicities and those belonging to traditional populations [12,13,14,15]. However, despite this progress, there is no clear consensus on host ethnicity and the field still suffers from inequity and lack of representation of many populations both within the United States and across the globe [16]. Additionally, it is generally thought that there is a substantial difference in microbial diversity and abundance across the dental arcade, the soft tissues of the mouth and throat, and of the saliva itself [17]. This diversity is also associated with the age of the individual [18]. Although there has been a concerted effort to address proper sample collection techniques across the oral cavity [19], they are not always ascribed to and, in many cases, individuals are not properly trained to collect difficult-to-access areas of the mouth, including periodontal pockets.

Outside of sampling type and host variation, individual dental care is the most addressable aspect of microbial diversity that may lead to dysbiosis. Periodontal disease is one of the global burdens of oral health, affecting 20–50% of the global population, and is predicated to increase in the future [20]. There are several forms, including the mildest (known as gingivitis), when gums become red, tender, and may bleed; mild to moderate periodontitis, when gums start to deepen and spaces between teeth increase; and moderate to advanced, which involves major bone or tissue loss [21]. The least severe form of periodontal disease, gingivitis, impacts about 50% of adults in the United States on an average of three to four teeth [22]. The oral microbiome has a critical influence on the presence of periodontal diseases, as the host immune–inflammatory response system reacts to the excess presence of supragingival plaque, thus exacerbating symptoms over time [23]. An additional issue is that many sufferers do not know the signs of disease progression and thus are unaware that they are impacted [24]. If enough time passes and the disease is untreated, surgical intervention may be necessary. However, various probiotic pastes and gums, along with ozone and chlorohexidine gels, have been posited as potential ways to maintain current gingival states and prevent recession [25,26]. Ethnicity is also thought to impact the presence of more severe forms of periodontal disease [27]. Hispanic populations in particular have the highest prevalence of periodontitis within the United States (63.5%) [28]. In addition to microbial dysbiosis within Hispanic periodontitis sufferers due to the disease itself, other factors such as alcohol consumption and smoking habits contribute to risk [29]. Other groups, such as non-Hispanic Blacks and non-Hispanic Asian Americans, also have high prevalences of periodontitis at 59.1% and 50.0%, respectively [28].

South Florida has undergone a population explosion, with a 7.4% rise in the last ten years according to the 2020 US Census, with an estimated 1.6% in the last year, making it the fastest growing state in the country [30]. In 2022, according to the Florida Department of Health, a total of 73.08% of Floridians identified as non-Hispanic (with 17.08% reporting as Black and 6.0% as any other ethnicity, including Asian) and 26.92% identifying as Hispanic [31]. South Florida, which generally refers to the Eastern tri-county area (Palm Beach, Broward, and Miami-Dade counties), saw an increase of 30,000 residents from 2022 to 2023, ranking ninth in the country in terms of most-populated metropolitan areas [32]. The research team on this project is from Nova Southeastern University (NSU) in Davie-Fort Lauderdale, Florida. It is a small private four-year university that is currently deemed an HSI (Hispanic Serving Institution), in that more than one-third of the university population identifies as Hispanic/Latino. Additionally, NSU has a large Asian and Black student population, with all self-identifying minorities comprising 62% of the total student population [33]. Thus, the research conducted here is primed for an opportunity to examine underrepresented diverse populations and those who may have an increased risk of susceptibility to this detrimental disease, even when patients hold a positive view of their own oral health.

Here, we examine a diverse ‘healthy’ South Florida campus population from a private university with a dental medicine program. Our goal is to examine how supragingival plaque microbes differ across different ethnic groups, thus potentially impacting future treatments of oral disease. We also assess within-individual diversity by collecting plaque from four groups of molars in different mouth quadrants (upper left, upper right, lower left, lower right) and from the tongue (scraping). Through collection, extraction, polymerase chain reaction (PCR) amplification, and 16S rRNA next generation sequencing (NGS), we compare microbial abundance, alpha diversity, beta diversity, and linear discriminant analysis effect size (LefSe) across samples. Microbial abundance is documented as the number of taxa tallied at the phyla and genera levels. Alpha diversity is a common measure used in microbiome studies to examine the extent of unique microbial taxa within a single sample, whereas beta diversity compares the extent to which taxa are shared between samples [34,35]. A LefSe analysis determines which taxa are most likely to explain differences between specific metrics [36]; in this case, a periodontally healthy oral cavity, localized gingivitis, and generalized gingivitis. We use these abundance, diversity, and linear discriminant analyses to arrive at conclusions associated with ethnicity, along with secondary metrics such as host sex, health status, and sampling location within the dental arcade.

## 2. Materials and Methods

Prior to any patient contact or sample collection, the study protocol was approved by the Institutional Review Board for Research with Human Subjects at Nova Southeastern University (IRB #: 2020-397-NSU). This institutional IRB acted in accordance with the 1964 Helsinki Declaration and all the later amendments and comparable standards of ethics. After initial screening over the phone to determine eligibility, written informed consent was obtained from all participants who provided samples.

Subjects were recruited using flyers posted around the Davie-Fort Lauderdale NSU campus and emailed to current students and graduate students. These flyers included contact information of the PI (ATO) who performed the initial screening and included the following criteria: age 18–40, resident of South Florida for >6 months, absence of active periodontal disease, and willing and able to give informed consent and participate in all aspects of this study. Individuals were excluded if they had uncontrolled medical conditions, had ever been diagnosed with periodontal disease, were pregnant or lactating, had used antibiotics in the last 3 months, were taking bisphosphonate or medications known to impact soft tissue inflammation, or had dental implants, and any NSU dentists, dental students, and dental hygienists. These requirements are commonplace within the NSU College of Dental Medicine research sector and thus were utilized for the purposes of this investigation. A total of 62 potential participants called the number on the flyer and left contact information to be reached regarding participation in this study. The PI contacted these individuals over the phone to conduct preliminary screening (these interviews were not recorded) and a total of 45 individuals were invited and appeared at their scheduled appointments, which allowed them to participate in the initial sampling collection phase of this study (Supplemental Table S1).

Invited participants arrived at the College of Dental Medicine facility and documented their age, sex, smoking status, BMI, and ethnicity. Patients were requested to not eat or drink anything other than water from the time they went to bed to the time they arrived at the facility in the morning (7 a.m. to 11:30 a.m.). Screened data were reviewed (SD and CG) and blood pressure was taken. The patient’s periodontal chart and oral and intra-oral exam were completed by SD in order to assess overall health, including probing depth, clinical attachment level, modified sulcus bleeding index, and plaque load. All of these metrics were used by the clinicians to determine the extent, if any, of periodontal disease [37]. Supragingival plaque samples were removed from premolars and molars in four quadrants of the mouth using a sterile Gracey curette (Hu-Friedy, Chicago, IL, USA) for each section: upper left, upper right, lower left, lower right, and tongue (scraped using a nylon mixing spatula (G&H Orthodontics, Franklin, IN, USA). Samples were placed into a tris-acetate-EDTA buffer (Thermo Fisher Scientific, Waltham, MA, USA) and frozen at −80 °C until extraction. Participants were compensated for their time with a USD 25 Target gift card.

Samples were thawed and extracted using a QIAamp BiOstic bacteremia DNA kit (Qiagen, Hilden, Germany) according to the manufacturer’s protocols. The resulting elution was quantified using a nanodrop photospectrometer (Thermo Fisher Scientific, Waltham, MA, USA) and samples below 5 ng/µL were omitted from downstream analysis. DNA underwent PCR amplification for the 16S rRNA (ribosomal) gene V4 region (515F and 806R) [38]. PCR reactions utilized Accustart PCR Toughmix II (Quantabio, Beverly, MA, USA) for 35 cycles in accordance with the manufacturer’s protocols. The mix for each sample only differed in the use of the forward 515F barcode. Samples were amplified in duplicate, then combined, and the success of the PCR was confirmed using a 2% SeaKem LE agarose (Lonza, Washington, DC, USA) gel electrophoresis with GelRed nucleic acid gel stain dye (BioTium, Fremont, CA, USA) at 100 mV for 45 min. Band intensity (darkness) was visually compared with the 100 base pair DNA ladder (Fisher Scientific) and pooled accordingly, with those samples exhibiting no bands being removed, light bands having 3 µL added, medium bands having 2 µL added, and dark bands having 1 µL added to the pool. Samples were purified initially using a MinElute PCR purification kit (Qiagen) and purified a second time using AMPure XP beads (Beckman Coulter, Brea, CA, USA) according to the manufacturer’s protocols. Fragment size and concentration was confirmed using an Agilent TapeStation (Agilent Technologies, Santa Clara, CA, USA) and Qubit 2.0 (Thermo Fisher Scientific). Samples were diluted to 4 nM and included a PhiX spike (15%) for quality control before being run on an Illumina MiSeq V3 600 cycle single-end chemistry (Illumina, Hayward, CA, USA) in the Molecular Microbiology and Genomics Laboratory at NSU.

Samples were retrieved from BaseSpace (Illumina, Hayward, CA, USA) following successful sequencing and imported into QIIME2 [39]. Samples were first demultiplexed, quality filtered (QC > 30), merged, and had chimeric reads removed and ends trimmed (29 base pairs on each side) using DADA2 [40]. Samples were compared with the SILVA rRNA database (Version SSU r138) in order to ascribe operational taxonomic units (OTUs) [41]. A total of 135 samples were sequenced (26 participants, 1 of whom was sampled twice) and 131 samples were used for subsequent analyses (4 samples were omitted due to <10,000 sequencing reads: FN08-T, FN26-T, FN33-T, FN47-UL). Details on participants and sequencing statistics can be found in Supplemental Table S1.

Taxonomic abundances were first rarefied to 10,000 reads, with the top 100 most common OTUs being visualized at the phyla and genus levels. Alpha diversity was presented using observed OTUs and significant differences were ascertained. Beta diversity was visualized as a weighted Bray–Curtis principal coordinate analysis. LefSe analyses were used to compare the dominant genera across periodontal health cohorts, i.e., healthy/localized, healthy/generalized, localized/generalized. All tests (unless noted) and visualizations used were visualized using ‘ggplot2’, ‘phyloseq’, ‘microbiome’, ‘microbiomeMarker’ and ‘microbiomeutilities’ packages within R (4.3).

## 3. Results

A total of 45 participants were invited to the facility. They consented, provided demographic information, and underwent dental examinations and sampling procedures. From the total of 45 subjects, a number of individuals did not meet the criteria to be included further in this study. This could have been due to any number of factors, including incomplete demographic information, eating or brushing prior to the morning sampling visit, undetectable DNA concentration in at least three of the sampled sites, non-amplification despite repeated PCR attempts, or DNA sequences not meeting quality or abundance thresholds (Supplemental Table S1). As such, a total of 26 individuals were included in this study, with 1 participant providing a sample twice, one year apart (54a and 54b). From that total, there were 9 females (35%) and 17 males (65%), who described themselves as multiethnic (12%), Hispanic (15%), Black (19%), Asian (27%), and Caucasian (27%). In terms of health, 6 individuals (23%) showed localized gingivitis, while 2 (8%) showed generalized gingivitis and 18 (69%) were deemed periodontally healthy after dental assessment. Regarding body mass index (BMI), 15 (58%) individuals exhibited BMI within the normal range, 4 (15%) were overweight, and 7 (27%) were classified as obese. Additional participant details can be found in Table 1.

A total of 8,698,149 de-multiplexed reads were recovered, with per-sample read counts ranging from 2489 to 203,787 reads. After duplicate, chimeric, and QC < 30 reads were removed, a total of 7,418,279 sequence reads were used for downstream analysis.

**Figure 1:**
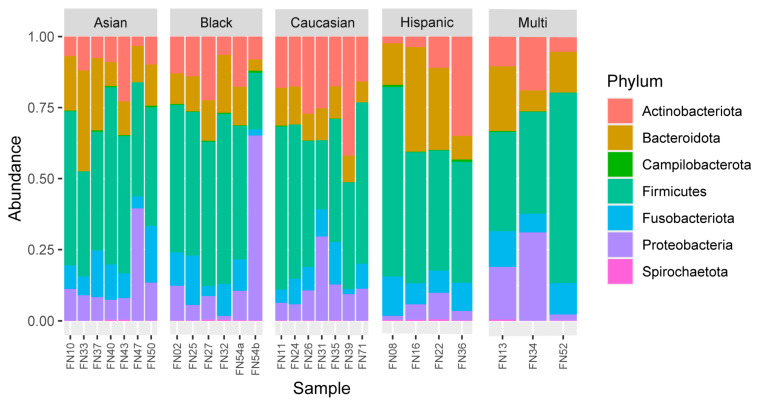
Bar graph reflecting the phyla abundance across all samples. The abundance shown here includes the top 100 most common OTUs after samples were first rarefied to 10,000 reads.

**Figure 2:**
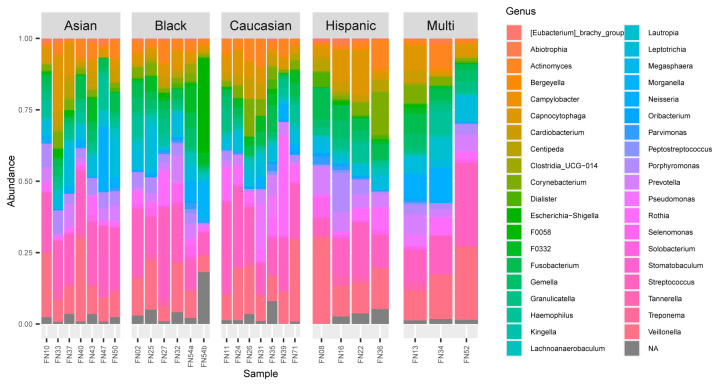
Bar graph reflecting the genus abundance across all samples. The abundance shown here includes the top 100 most common OTUs after samples were first rarefied to 10,000 reads.

The most common phyla found across samples were Firmicutes (46.7%), Bacteroidota (15.2%), Actinobacteriota (14.9%), Proteobacteria (13.1%), and Fusobacteriota (9.5%). The distribution of these phyla across all samples (all sites combined for each individual) can be seen in Figure 1. The top abundance within genera for samples included Streptococcus (18.1%), Veillonella (14.1%), Capnocytophaga (5.4%), and Leptotrichia (5.2%), followed by Prevotella and Fusobacterium (both 4.2%). The distribution of these genera across all samples (all sites combined for each individual) can be seen in Figure 2.

**Figure 3:**
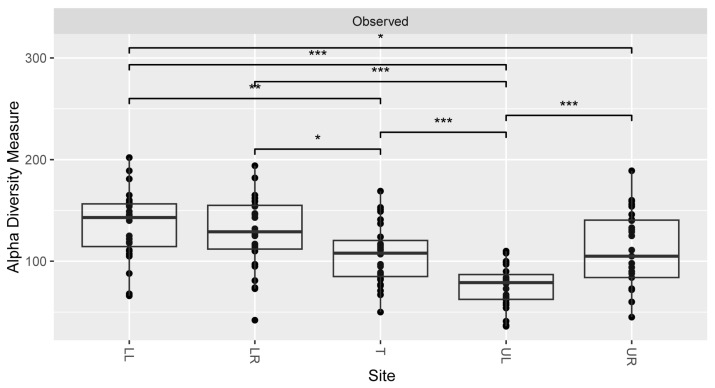
Alpha diversity bar plot reflecting the total observed unique OTUs across each of the five sampling locations (LL—lower left, LR—lower right, T—tongue, UL—upper left, UR—upper right). Significance values were documented (* *p* < 0.05; ** *p* < 0.01; *** *p* < 0.001) and all samples were rarefied to 10,000 reads.

**Figure 4:**
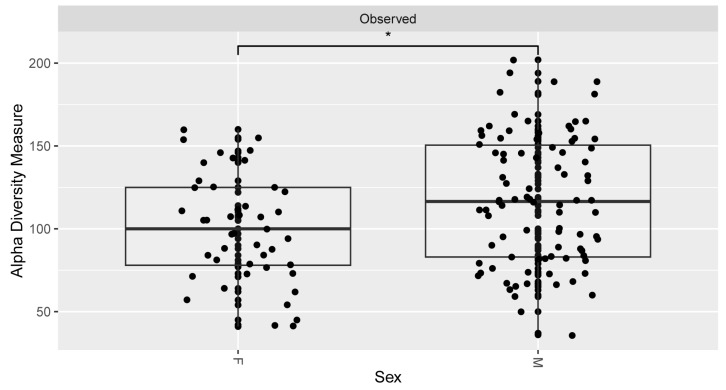
Alpha diversity bar plot reflecting the total observed unique OTUs across each sex (M—male, F—female). The results between the two sexes were significant (at the * *p* < 0.05 level) and all samples were rarefied to 10,000 reads.

Alpha diversity metrics (observed OTUs) showed the most diversity within the lower left quadrant and the least diversity in the upper left quadrant (Figure 3). There were statistically significant differences between several areas of the mouth, i.e., lower right and tongue, upper right and lower left (* *p* <0.05), tongue and lower left (** *p* < 0.01), and upper left with all four other quadrants (*** *p* < 0.001). Additionally, females had a lower alpha diversity than males based on the same metrics (*p < 0.05) (Figure 4). No other significant values at alpha diversity were detected based on any other demographic or health information.

**Figure 5:**
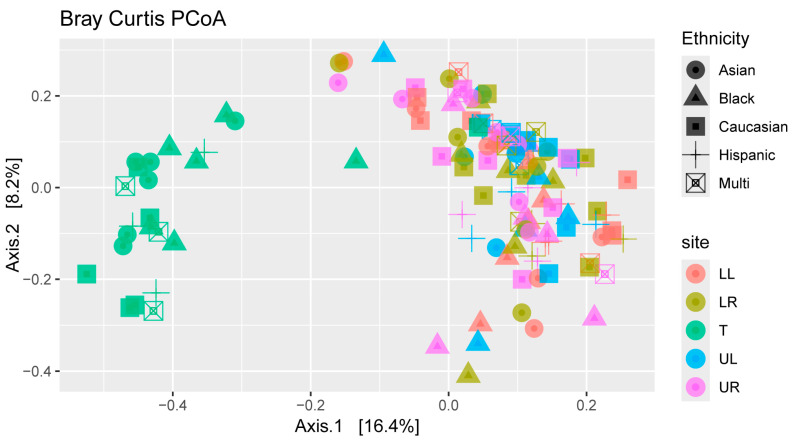
Beta diversity plot reflecting weighted Bray–Curtis principal coordinate analyses involving samples rarefied to 10,000 reads. Each point is associated with a single sample, with the shape indicating ethnicity and the color indicating sampling location (LL—lower left, LR—lower right, T—tongue, UL—upper left, UR—upper right).

A weighted Bray–Curtis principal coordinate analysis was used as a beta diversity metric across all samples (Figure 5). Although no clear clustering was visualized across different ethnicities, samples from the tongue were clustered along the left side of axis 1 (describing 16.3% of the variation) and spread vertically across axis 2 (describing 8.2% of total variation).

**Figure 6:**
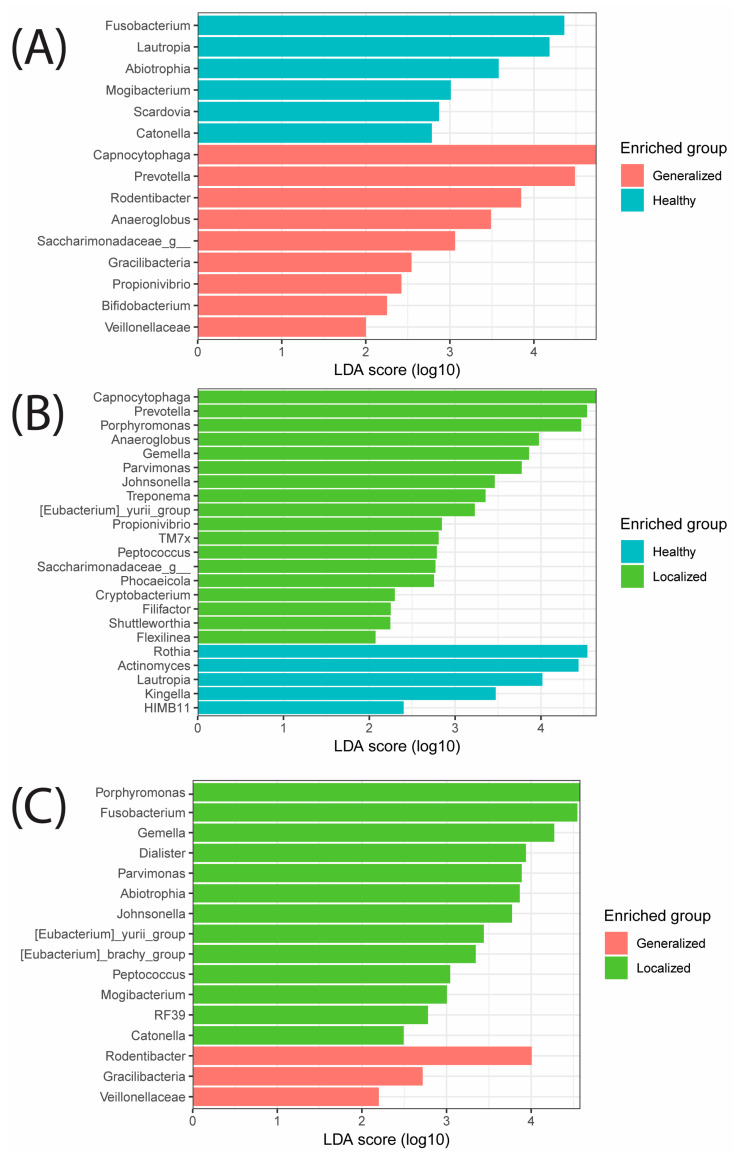
LefSe analysis showing the enriched groups of bacterial genera associated with the three periodontal health statuses: (**A**) generalized gingivitis and a periodontally healthy oral cavity, (**B**) localized gingivitis and a periodontally healthy oral cavity, and (**C**) generalized gingivitis and localized gingivitis.

Lastly, a LEfSe was used to identify differences between individuals with good periodontal health status, localized gingivitis (<20% of teeth), or generalized gingivitis (>20% of teeth), as seen in Figure 6. When periodontally healthy subjects were compared with gingivitis patients, periodontally healthy subjects had genera from Fusobacterium and Lautropia enriched, while generalized gingivitis subjects had Capnocytophaga and Prevotella enriched (Figure 6A). Periodontally healthy mouths also had Rothia and Actinomyces genera enriched compared with localized gingivitis subjects who had Capnocytophaga and Prevotella enriched, as seen with generalized gingivitis (Figure 6B). When compared with two dysbiotic health statuses, localized gingivitis had more enriched genera, with Porphyromonas and Fusobacterium leading, whereas generalized gingivitis had genera from Rodentibacter and Gracilibacteria enriched (Figure 6C).

## 4. Discussion

Overall, the reported phyla and genera detected in our population of South Floridians was in line with many oral studies of the same nature. However, we observed some significant differences in the number of observed bacteria between males and females and between many regions of the mouth, with the upper left quadrant, surprisingly, being the least diverse of all areas, including the tongue. We saw that the types of bacteria on the tongue separated when visualized with an unweighted PCoA, but the same did not factor for host ethnicity. Lastly, through linear discriminate analyses, we saw some differences in genera associated with periodontal healthy individuals and those with localized or generalized gingivitis.

Firmicutes and Bacteroidota phyla dominate the healthy human oral cavity and other locations such as the human gut [42,43]. In fact, the five most common phyla documented in this study have been consistently found as the most common in studies for some time [44,45]. Although likely a true finding, one of the reasons these phyla continue to be common may be due to database biases [46]. Firmicutes and Bacteroidota have been postulated to be part of an alliance against the detrimental genera within the Actinobacteria phyla, thus protecting the overgrowth of opportunistic pathogens [47]. Many Fusobacteria, like Firmicutes and Bacteroidota, are considered to be symbiotic within the human oral cavity [48]. Proteobacteria presence is known to be a signature of dysbiosis within the human gut [49], but their role within the oral cavity is less clear.

The most common bacterial genus seen within the NSU South Florida population is Streptococcus. Streptococcus is considered an aerobic colonizer, occupying the mouth early in life, along with other documented genera such as Actinomyces, Neisseria, and Veillonella [2]. They are an important part of biofilm production, so it is not surprising that their presence within supragingival plaque was documented here [50]. This also holds true for Veillonella, in that many species are more commonly found within supragingival and subgingival plaque, while others are more commonly found on the tongue dorsum, tonsils, throat, and hard palate [51]. Prevotella was previously found in underrepresented populations and thought to be a potentially harmful pathogen [12] and likely reached beyond just the oral cavity, having systemic impacts on the human body [52]. Like Prevotella, Capnocytophaga abundance is also considered to impact host health but in a more localized way; studies have shown it is associated with poor oral hygiene and periodontal inflammation [53]. Leptotrichia has also been shown to be present in patients with gingivitis [53]. The results reported here can lead to mixed conclusions, as most of the South Florida cohort was assessed as orally healthy. Some of the most dominant genera seen are commonly thought of as drivers of dysbiosis and disease states, thus further confounding their actual roles within a healthy cohort.

Alpha diversity plots in our study showed information about the distribution of different types of bacterial species across different sample types. Our results showed significant differences in the number of bacterial types present within locations in the mouth. Specifically, the tongue and upper left quadrants were found to be significantly different from most of the other quadrants. However, the even lower diversity of microbes within the upper left quadrant of the mouth was less clear. We postulate that it had something to do with the handedness of the individuals (which unfortunately was not documented), although even that can be misleading, since, depending on the study, it has been documented that both right [54]- and left [55]-handed individuals have better oral hygiene. Brushing patterns could also be implicated, along with preferred chewing side [56]. In our study, females were found to have a lower alpha diversity, which follows considering that females are thought to have better dental hygiene than men [57]. We can speculate that excessive toothbrushing may lead to less microbial abundance overall and thus lower diversity in healthy individuals. Alpha diversity differences at the sex level may also be impacted by host hormones [58].

Based on beta diversity plots, the tongue samples clustered together in a separate area compared with the rest of the plaque samples. This was not a surprise, as the tongue harbors both a different type of ecology and, according to the alpha diversity, a less robust ecology [59]. These differences in microbial types in the tongue is associated mainly with its role in the circulation of aerobic microbiota within the saliva; although, papillae within the tongue itself can contain some anerobic sites [2]. The circulation of saliva within the mouth compared with the biofilm-encased microbiota in gingival sulci is likely the root cause of the differences between tongue and teeth. Although we attempted to sample participants in the morning, the time of day of the sample collection may also have implicated the presence of certain microbes [60].

Although studies have repeatedly shown that the host culture and geography impact the oral microbiome [11], our beta diversity plot did not show any clustering based on ethnicity. Although the majority of participants in our study self-identified as Caucasian, we were able to obtain a random sample of primarily college-aged students from diverse backgrounds using only voluntary recruiting methods. Essentially, everything is viewed as impacting the human microbiome, including age and diet [18,61,62], but, even with this in mind, we did not see evidence of partitioning based on the ethnic background of the participants.

Lastly, a LEfSe analysis revealed a clear association with certain genera related to periodontal health and gingivitis-impacted individuals. Both the generalized and localized gingivitis groups showed the same top two genera (Capnocytophaga and Prevotella) enriched compared with the healthy subset. As previously mentioned, both of these genera were present in the top five abundances across all samples, which suggests that perhaps the abundance of these genera are the driving factors of oral dysbiosis related to gingivitis, something that has been shown in previous studies [63]. This supports a previous study that found Capnocytophaga present in 79% of a sample of 300 individuals, but was also more associated with gingivitis sufferers [64]. Prevotella seems to behave in a similar manner in that it is a common oral microbe, but particular species and abundances can be a root cause of oral disease [52]. In future studies, for those who choose to focus on periodontal health, we recommend examining more proactive treatments such as probiotics and ozonized substances, which have shown promise in slowing disease progression [25,26]. Additionally, as was the main goal of this study, we hope researchers can address ethnicity-specific oral health issues in diverse communities such as in South Florida.

A number of challenges arose with this study. Once funding was obtained, recruiting was set to begin in July 2020, during the first months of the COVID-19 pandemic. Although the dental clinic at NSU continued to operate, recruitment process slowed and stretched over several years, to the point that one individual showed up and donated samples twice, thinking it was a separate study altogether. The team was a small one and, at times, information was omitted by the patient and not initially detected, or information obtained during the oral health assessment by the dentist was incorrectly coded by the resident assistant. For the sake of the integrity of this study, any missing or perhaps incorrectly input information caused that participant to be removed, thus reducing the number of individuals.

These data were intended to act as pilot information for a larger study of the impact of host ethnicity on the abundance and diversity of oral microbiota. In the future, we hope to address questions of brushing habits (handedness, frequency, electric vs. manual toothbrush use), plaque load, and, interestingly, the impact of long COVID-19 infection on microbiota in individuals from varying backgrounds. We also hope to undertake case control studies related to periodontal health of underrepresented groups, potentially integrating probiotic and ozone and chlorohexidine gels as interventions.

## 5. Conclusions

Despite assembling a diverse healthy university cohort in South Florida, we did not see significant differences in microbial diversity related to our main objective metric of host ethnicity. We did, however, see some associations with host sex and sampling location within the mouth. Additionally, although free of periodontitis, several of our participants had localized or generalized gingivitis, which, based on previous research, is not unsurprising. The genera of bacteria present within those individuals showed an increased association with the healthy and diseased states. This research stresses the overwhelming complexity of the oral ecosystem and encourages stringent screening and data collection protocols to ensure reliable results. Lastly, our work emphasizes the continued inclusion of underrepresented groups in oral microbiome studies moving forward.

## Figures and Tables

**Table 1:** Details of the participants in this study.

Sample	Age Range	Age	Ethnicity	Sex	BMI_range	Height (in)	Weight (lbs)	BMI	Health State
FN02	<22	20	Black	M	overweight	77	199	34.15	Healthy
FN08	<22	27	Hispanic	M	overweight	71	185	25.8	Localized
FN10	23–27	27	Asian	F	normal	61	100	18.89	Localized
FN11	<22	20	Caucasian	M	overweight	71	180	25.1	Healthy
FN13	<22	24	Multi (Caucasian, Asian)	M	overweight	68	167	25.39	Healthy
FN16	<22	22	Hispanic	M	obese	72	250	33.9	Localized
FN22	33–38	20	Hispanic	M	overweight	69	190	28.06	Localized
FN24	28–32	21	Caucasian	F	normal	65	139	23.13	Generalized
FN25	<22	38	Black	M	obese	76	250	30.43	Healthy
FN26	33–38	30	Caucasian	F	obese	48	145	44.24	Healthy
FN27	33–38	18	Black	F	normal	60	125	24.41	Healthy
FN31	28–32	35	Caucasian	M	normal	73	155	20.45	Healthy
FN32	28–32	33	Black	F	normal	67	135	21.14	Localized
FN33	<22	30	Asian	M	normal	70	134	19.22	Localized
FN34	33-38	29	Multi (Caucasian, Hispanic)	M	normal	70	170	24.39	Healthy
FN35	28–32	22	Caucasian	M	normal	69	145	21.41	Healthy
FN36	28–32	37	Hispanic	F	overweight	64	164	28.15	Healthy
FN37	<22	29	Asian	M	normal	73	160	21.11	Generalized
FN39	33–38	31	Caucasian	F	normal	68	140	21.28	Healthy
FN40	<22	20	Asian	M	normal	64	135	24.03	Healthy
FN43	<22	33	Asian	M	normal	67	130	20.36	Healthy
FN47	23–27	19	Asian	M	normal	71	160	22.31	Healthy
FN50	23–27	19	Asian	F	normal	64	135	23.17	Healthy
FN52	23–27	25	Multi	M	overweight	67	230	36.02	Healthy
FN54a	23–27	26	Black	M	obese	72	400	54.24	Healthy
FN54b	23–27	26	Black	M	obese	72	400	54.24	Healthy
FN71	23–27	23	Caucasian	F	normal	66	125	20.17	Healthy

## Data Availability

Raw data will be available through the NCBI Short Read Archive under Bioproject PRJNA1154284 and BioSamples SAMN43410097 to SAMN43410231. QIIME2 Conda (2022.2) and R code can be found at the GitHub site of user atozga.

## Supplementary Materials

The following supporting information can be downloaded at: https://www.mdpi.com/article/10.3390/microorganisms12091921/s1, Supplemental Table S1: This table provides detailed sequencing statistics for those participants included in the study. It also provides information on those who were omitted from the study for a variety of reasons.
